# The POSEIDON stratification - moving from poor ovarian response to low prognosis

**DOI:** 10.5935/1518-0557.20200100

**Published:** 2021

**Authors:** Matheus Roque, Thor Haahr, Sandro C. Esteves, Peter Humaidan

**Affiliations:** 1 MATER PRIME - Reproductive Medicine, São Paulo, SP, Brazil; 2 The Fertility Clinic Skive Regional Hospital, 7800 Skive, Denmark; 3 Faculty of Health, Aarhus University, 8000 Aarhus C, Denmark; 4 ANDROFERT, Andrology and Human Reproduction Clinic, Campinas, SP, Brazil

**Keywords:** POSEIDON, poor ovarian response, Bologna criteria, low-prognosis patients, IVF/ICSI, assisted reproduction

## Abstract

Poor ovarian response remains one of the most challenging tasks for an IVF clinician. In this review, we aim to highlight the ongoing research for optimizing the prognosis in poor ovarian response patients. The newly introduced POSEIDON criteria argue that the first step is to move from a poor response to a poor prognosis concept, while improving identification and stratification of the different sub-types of poor prognosis patients prior to ovarian stimulation. The immediate marker of success is the ability of the ovarian stimulation to retrieve the number of oocytes needed to obtain at least one euploid blastocyst for transfer in each patient. This surrogate marker of success should not replace live birth as the most important outcome, but it should be approached as a useful tool for clinicians to evaluate their strategy for achieving live birth in the shortest timespan possible in the individual patient/couple.

## INTRODUCTION

It is estimated that more than 1.5 million *in vitro* fertilization (IVF)/intracytoplasmic sperm injection (ICSI) cycles are performed every year around the world, and the number of initiated IVF cycles increases by almost 9.5% per year (Dyer *et al*., 2016). Ovarian stimulation (OS) - the first step in every IVF/ICSI cycle− is crucial to increase IVF outcome, as the development of a sufficient number of follicles and oocytes increase not only the live birth rate per cycle, but, importantly, also the cumulative live birth rate (CLBR) per cycle in ART treatments (Drakopoulos *et al*., 2016; Siristatidis *et al*., 2013). Indeed, the number of oocytes retrieved during an IVF treatment is of utmost importance to overcome two critical problems related to female infertility, namely, oocyte competence and ovarian aging.

In regards to oocyte competence, the live birth rate per mature oocyte retrieved is lower than 5% (Goldman *et al*., 2013), and the number of oocytes needed to obtain at least one live birth increases exponentially with age (Goldman *et al*., 2017). This increase in the number of oocytes necessary to obtain a live birth is associated with the biological aging of the ovaries, resulting in a progressive decrease in embryo euploidy rates with increasing age (Esteves *et al*., 2019a).

Importantly, when evaluating OS outcomes, a significant number of patients - ranging from 9% to 24%− exhibit poor ovarian response (POR) to stimulation with exogenous gonadotropins. As a consequence, low pregnancy and live birth rates are obtained, varying from 3% to 14% (Tarlatzis *et al*., 2003; Ulug *et al*., 2003; Polyzos *et al*., 2012; 2013; La Marca *et al*., 2015; Drakopoulos *et al*., 2016; Humaidan *et al*., 2017).

The clinical management of the POR patient is challenging, as there is no single intervention, which seems to clearly improve IVF outcomes for this subgroup of patients (Papathanasiou *et al*., 2016). Although several strategies have been proposed to optimize the ovarian response and the number of oocytes retrieved, currently there is no consensus regarding the most optimal treatment for the POR patient undergoing IVF (Pandian *et al*., 2010; Papathanasiou *et al*., 2016, Olgan & Humaidan, 2017). Moreover, the diversity in the definition of POR introduce significant limitations in interventional trials, as it is likely that patients with different characteristics are compared (Polyzos & Devroey, 2011; Papathanasiou *et al*., 2016).

The Bologna criteria (2011) were published in an attempt to standardize the definition and diagnosis of POR, and to compare results as well as to draw reliable conclusions in a more homogeneous population (Ferraretti *et al*., 2011). According to these criteria, for a woman to be classified as a POR patient, at least two of the following three criteria must be present, namely, (i) Advanced maternal age (≥ 40 years) or any other POR risk factor; (ii) Previous poor ovarian response (≤3 oocytes retrieved or previous cycle cancelled), and (iii) Abnormal ovarian reserve tests (antral follicle count [AFC] < 5-7 follicles or Anti-Mullerian hormone [AMH] < 0.5-1.1 ng/ml). Moreover, two episodes of POR after maximal stimulation were deemed sufficient to classify a patient as POR - even in the absence of the other criteria mentioned (Ferraretti *et al*., 2011). Although the development of the Bologna criteria focused on establishing homogeneous subgroups of patients considered as POR, heterogeneity was still a major problem within the Bologna criteria group (Humaidan *et al*., 2017; Bozdog et al., 2017; Esteves *et al*., 2018). Moreover, the Bologna criteria failed to assess the reproductive potential of young POR patients (Cohen *et al*., 2015; Alviggi *et al*., 2018; Boots & Bernardi., 2018).

Notably, there are significant differences in the reproductive outcomes when evaluating patients within different age categories; thus, emphasizing the importance of taking into account quantity as well as the quality of oocytes (Bozdag *et al*., 2017). Hu *et al*. (2014) retrospectively evaluated 592 IVF cycles in patients classified according to the Bologna criteria, comparing the outcomes of different ovarian stimulation protocols. Although, not all subgroups of the Bologna criteria were analyzed, there were different reproductive outcomes when comparing patients below and above 35 years of age. In their study, the mean implantation rates ranged from 15.3% to 29.4% in patients below 35 years, and from 6.3% to 24.1% in patients ≥35 years (Hu *et al*., 2014). Bozdag *et al*. (2017) conducted a retrospective evaluation of the IVF outcomes of 821 patients who fulfilled the Bologna criteria, and for whom 1,257 ICSI cycles was performed. In this study, the live birth rates were lower than 10% overall. However, the authors reported differences in results within the Bologna Criteria subgroups, concluding that the subgroups were non-comparable in terms of reproductive potential (Bozdag *et al*., 2017).

### POSEIDON - and the concept of low prognosis

To overcome the heterogeneity observed within the different groups classified as POR following the Bologna criteria, the POSEIDON (acronym for Patient-Oriented Strategies Encompassing IndividualizeD Oocyte Number) criteria were developed, moving from a poor ovarian response concept to a low prognosis concept. This new concept was introduced to stratify low prognosis patients undergoing ART based on the combination of quantitative and qualitative parameters, proposing a new and more detailed stratification of low prognosis IVF patients (Alviggi *et al*., 2016). The novel concept of low prognosis −defined in terms of CLBRs per initiated cycle− focusses on improving the management of patients undergoing ART by identifying a more homogeneous population and by suggesting a tailored approach to patient handling, and, thus, providing better tools to maximize IVF success rates. In this review, we aim at presenting the four groups stratified according to the POSEIDON criteria; evaluate their reproductive prognosis and the suggested clinical handling of each group, in an effort to establish the best strategy to improve the reproductive outcomes of the low prognosis patient undergoing an IVF treatment.

### Oocyte quantity and cumulative live birth

The ovarian response and the number of oocytes retrieved after OS are independent predictors of the likelihood of a live birth during an IVF treatment (Sunkara *et al*., 2011; Drakopoulos *et al*., 2016). The CLBR per cycle markedly increases as the number of oocytes retrieved increases (Polyzos *et al*., 2018). In this line, Drakopoulos *et al*. (2016) reported that the odds ratio (OR) for CLBR significantly increases with the number of oocytes. When comparing the group of patients who had 0-3 oocytes retrieved, patients with 4-9 oocytes had an OR of 2.4 (95% confidence interval [CI] 1.3-4.4), 10-15 oocytes an OR of 3.5 (95% CI 1.9-6.7), and >15 oocytes an OR of 5.6 (95% CI 3.1-11.6). The group of patients with 4-9 oocytes, which was previously classified as normal, was renamed as suboptimal responders, as the CLBR per initiated cycle was poorer when compared to patients with 10 or more oocytes (Drakopoulos *et al*., 2016). Thus, concerning the number of oocytes retrieved, two subgroups of patients presented with poorer clinical outcomes, namely, those with <4 oocytes retrieved (poor response) and those with 4-9 oocytes (suboptimal response). However, these groups of patients are likely to have different characteristics, as the ovarian reserve, age, and the ovarian response to the treatment may differ among them, and consequently have an impact on CLBR.

Hence, the ‘low prognosis’ concept fundamentally relates to CLBR, which is defined by the International Committee for Monitoring Assisted Reproductive Technologies (ICMART) as, ‘*the number of deliveries with at least one live birth resulting from one initiated or aspirated ART cycle, including all cycles in which fresh and/or frozen embryos are transferred, until one delivery with a live birth occurs or until all embryos are used, whichever occurs first, expressed per 100 cycles (initiated or aspirated)*’ (Zegers-Hochschild *et al*., 2017).

### POSEIDON stratification

**Figure 1:**
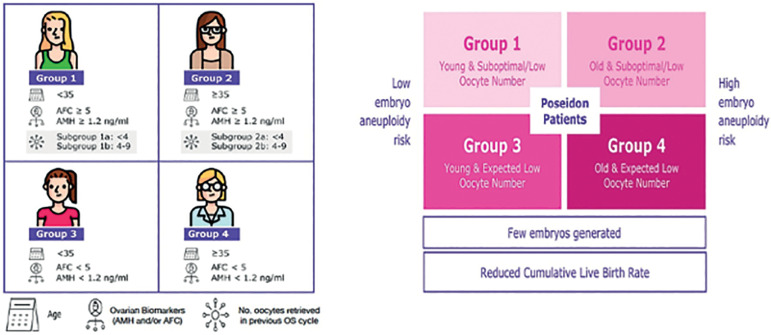
POSEIDON criteria of low prognosis patients in ART. Art drawing by Chloé Xilinas. Modified from Esteves et al. *Front. Endocrinol.* 2019;10:814. (This is an open-access article distributed under the terms of the Creative Commons Attribution License (CC BY).

The novel system relies on female age, ovarian reserve markers, ovarian sensitivity to exogenous gonadotropin, and the number of oocytes retrieved, which will both identify the patients with low prognosis and stratify such patients into one of four groups of women with “expected” or “unexpected” impaired ovarian response to appropriate exogenous gonadotropin stimulation. According to these criteria, four distinct groups of low prognosis patients can be established. Group 1 - patients <35 years with adequate ovarian reserve parameters (AFC>=5 or AMH>=1.2 ng/mL), presenting with an unexpected poor (<4 oocytes retrieved - Subgroup 1a) or a suboptimal (4-9 oocytes retrieved - Subgroup 1b) ovarian response after OS; Group 2 - patients >= 35 years with adequate ovarian reserve parameters (AFC>5 or AMH >=1.2ng/mL), presenting with an unexpected poor (<4 oocytes retrieved - Subgroup 2a) or a suboptimal (4-9 oocytes retrieved - Subgroup 2b) ovarian response; Group 3 - patients <35 years with poor ovarian reserve parameters (AFC <5 or AMH<1.2ng/mL); Group 4 - patients >=35 years with poor ovarian reserve parameters (AFC <5 or AMH<1.2ng/mL) (Alviggi *et al*., 2016; Humaidan *et al*., 2016; Esteves *et al*., 2018) - Figure 1 (left). Owing to low oocyte numbers and less embryos produced, POSEIDON patients have lower cumulative live birth rates per started cycle than non-POSEIDON counterparts. However, the prognosis is differentially affected by female age as it relates to the risk of embryo aneuploidy - Figure 1 (right).

The POSEIDON stratification is based on the age of the woman, ovarian reserve biomarkers, ovarian sensitivity to exogenous gonadotrophins, and the number of oocytes retrieved during an IVF cycle (if the patient underwent a previous OS), in order to stratify patients with a low reproductive prognosis. Thus, this novel stratification considers both quantitative and qualitative parameters of the patient, and include a new and more detailed stratification system of the infertile patient with “expected” and “unexpected” impaired ovarian response to exogenous gonadotropin stimulation (Alviggi *et al*., 2016). Importantly, POSEIDON groups 1-4 overall constitute approximately 47% of patients who undergo an IVF treatment (Conforti *et al*., 2019a).

**Figure 2:**
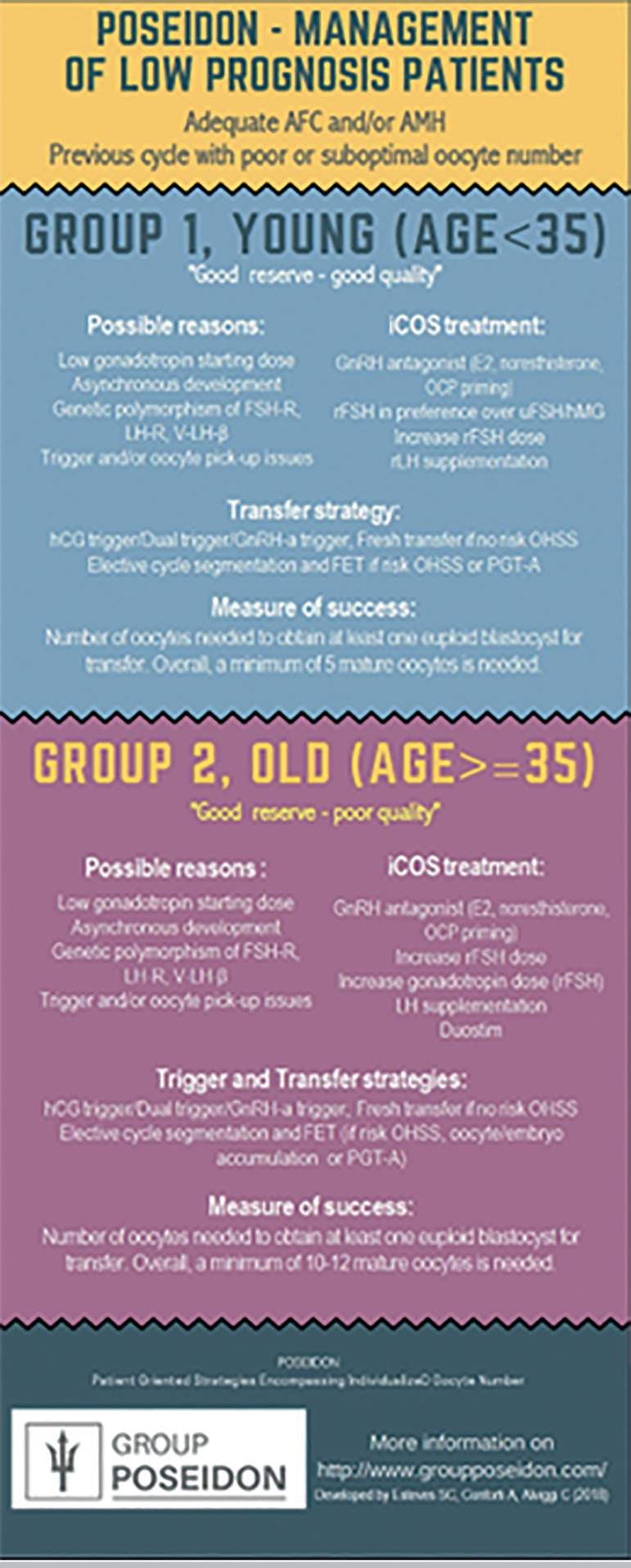
Management overview of low prognosis patients according to POSEIDON groups 1 and 2. Reprint from: Conforti et al. Management of Women With an Unexpected Low Ovarian Response to Gonadotropin. Front Endocrinol. 2019;10:387. This is an open-access article distributed under the terms of the Creative Commons Attribution License (CC BY).

In this stratification, POSEIDON groups 1 and 2 patients are those with an adequate ovarian reserve before treatment, but who had a low response to ovarian stimulation in terms of fewer follicles developed and fewer oocytes retrieved than expected from the ovarian reserve biomarkers; thus, leading to a lower CLBR per initiated cycle (Conforti *et al*., 2019a; Esteves *et al*., 2019b). The main hypotheses of this suboptimal response or “hypo-response” to OS are as follows, (i) polymorphisms related to the FSH and LH receptor, or polymorphisms related to circulating endogenous LH; (ii) suboptimal dosing of gonadotropins; (iii) Asynchronous follicular development during the OS; (iv) technical issues related to ovulation trigger and/or oocyte pickup. In accordance with these hypotheses, future stimulation strategies were suggested (Conforti *et al*., 2019b) - Figure 2.

**Figure 3:**
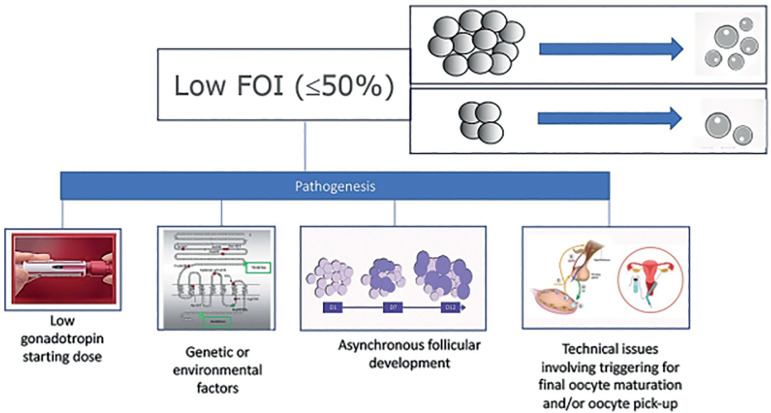
Pathogenesis of low follicle-to-oocyte index (FOI). Reprint from Alviggi et al. Understanding Ovarian Hypo-Response to Exogenous Gonadotropin in Ovarian Stimulation and Its New Proposed Marker- The Follicle-To-Oocyte (FOI) Index. Front Endocrinol. 2018; 9:589. This is an open-access article distributed under the terms of the Creative Commons Attribution License (CC BY).

The concept of a hypo-response to OS can be estimated by the FORT (follicle output rate) and FOI (follicle-to-oocyte index) (Conforti *et al*., 2019b). These indices correlate the pool of antral follicles at the beginning of OS to the number of preovulatory follicles at the end of stimulation - FORT (Genro *et al*., 2011; Gallot *et al*., 2012), or the number of oocytes retrieved at oocyte pickup - FOI (30). It has been considered that a FORT < 50% or a FOI <50% are suggestive of a hypo-response to stimulation, and subsequently specific strategies should be implemented in the next cycle to overcome the hypo-response (Conforti *et al*., 2019b) - Figure 3.

**Figure 4:**
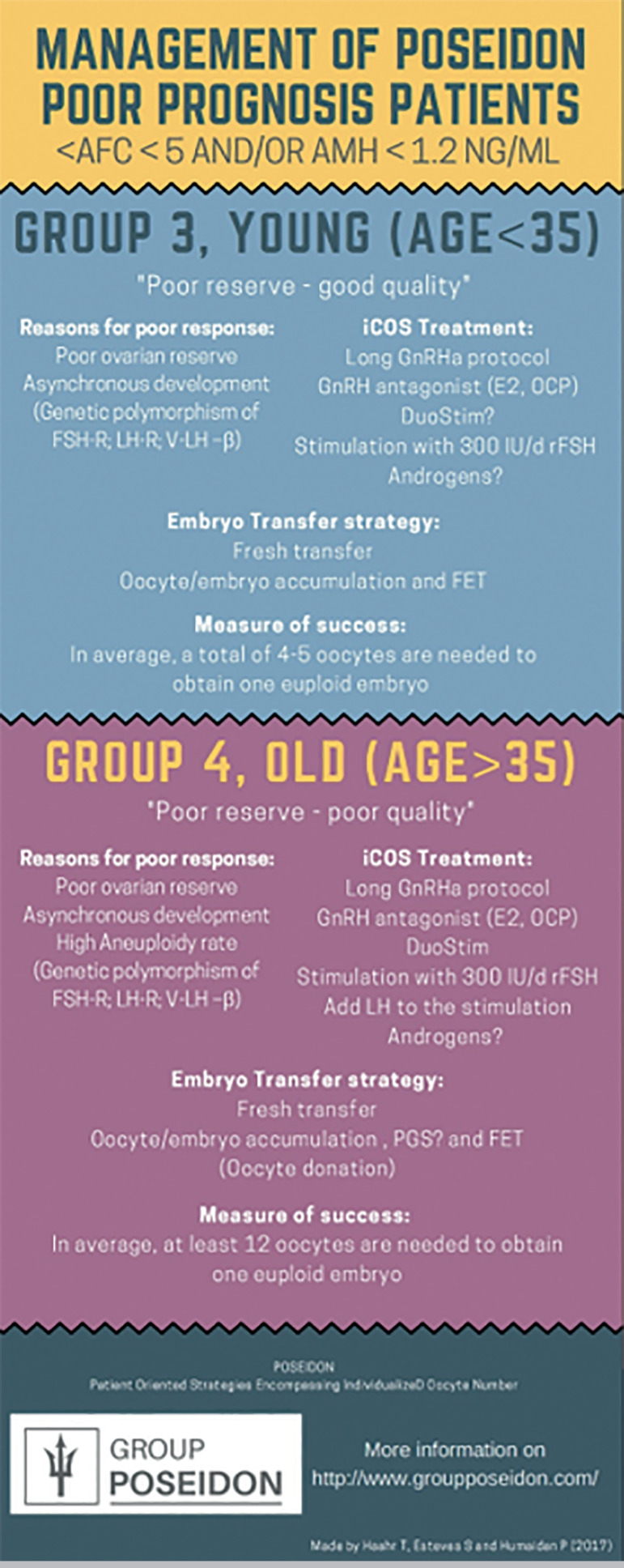
Overview of low prognosis patients according to POSEIDON groups 3 and 4, including pathogenesis and treatment options. Reprint from: Haahr et al. Individualized controlled ovarian stimulation in expected poor-responders: an update. Reprod Biol Endocrinol. 2018;16:20. This is an open-access article distributed under the terms of the Creative Commons Attribution License (CC BY).

POSEIDON groups 3 and 4 patients are patients with a poor ovarian reserve, in whom a poor ovarian response to stimulation is expected during their first IVF cycle (Haahr *et al*., 2019) - Figure 4. This fact per se gives them a high risk of a poor reproductive outcome, rendering clinical handling more challenging than POSEIDON groups 1 and 2 (Conforti *et al*., 2019b). With the current worldwide delay in childbearing, POSEIDON group 4 is increasingly being observed during IVF, constituting more than 50% of the total POSEIDON population in some centers, whereas group-3 patients constitute approximately 10%, only (Haahr et al., 2018a; 2018b; Haahr *et al*., 2019).

### Prognosis among different POSEIDON groups

When comparing the reproductive outcomes among different POSEIDON groups, a significantly different prognosis is seen in terms of CLBRs, and different treatment strategies should be implemented to change the fate of these patients. Recently, Leijdekkers *et al*. (2019) used the data from a multicenter observational study (OPTIMIST study) (Van Tilborg *et al*., 2017a; 2017b; Oudshoorn *et al*., 2017) to compare the prognosis of patients stratified according to the POSEIDON criteria. The authors concluded that individualizing the dose of gonadotrophins to be used during OS based on AFC did not increase CLBR when compared to non-individualization, using a 150 IU fixed daily dose of gonadotropins (Van Tilborg et al., 2017a; 2017b; Oudshoorn *et al*., 2017). Moreover, it was emphasized that the differences in prognosis among the four POSEIDON groups were based mainly on age and that the number of retrieved oocytes had little impact on CLBR. However, several researchers for a multitude of reasons, minimizing the clinical relevance of the findings (Haahr et al., 2018a; 2018b; La Marca et al., 2018; Sunkara & Polyzos, 2018), heavily criticized the OPTIMIST study. In contrast to the original OPTIMIST study, the study by Leijdekkers *et al*. (2019) reports staggering numbers underlining that non-individualization is detrimental to patients, when considering CLBR per treatment cycle. In the study by Leijdekkers *et al*. (2019), among the 985 women randomized to receive 150 IU FSH daily, 782 would be expected to have an adequate ovarian response, based on AMH levels. However, alarming levels of hypo-response to ovarian stimulation were identified, since 360 women of the 782 (46%) had a sub-optimal number of oocytes retrieved, i.e., <= 9 oocytes, and could be classified as POSEIDON 1 or 2. A normal response to treatment (10-15 oocytes) was seen in 30.1%, only, (242/782) and a hyper-response (>15 oocytes) in 23% (180/782) of all patients.

Thus, from these data revealing a high incidence of hypo-responses after OS, it is clear that patients would have benefitted from an individualized gonadotropin dosing. Moreover, Leijdekkers *et al*. (2019) concluded that the CLBR of the low-prognosis patient was approximately 56% over 18 months, and varied between POSEIDON subgroups, which was primarily attributed to the impact of age. However, after carefully evaluating data and the supplemental material, it becomes obvious that not only female age, but also the ovarian response to treatment have an impact on cumulative reproductive outcomes (Esteves *et al*., 2019c). This conclusion can be reached by comparing the different CLBRs per cycle in patients having a normal response to those classified as POSEIDON groups 1 to 4. Patients who had a normal response to OS achieved a higher CLBR per cycle when compared to patients with low prognosis according to the POSEIDON stratification. Thus, while the normal responder group of that study had a CLBR of 52% per cycle, POSEIDON groups 1, 2, 3 and 4 patients had CLBRs of 39%, 20%, 29%, and 17%, respectively. These differences in reproductive outcomes are clinically highly relevant, even though in the study mentioned above they were not statistically different, most probably due to the small sample size, which might have resulted in an imprecise estimate of the treatment effect (Stocking *et al*., 2019).

Furthermore, although the OPTIMIST study concluded that individualization of stimulation, based on the ovarian reserve of the patient did not add benefits vis-a-vis the CLBR after 18 months of treatment, Leijdekkers et al. (2019), using the database from the OPTIMIST study, showed that the lack of OS individualization in IVF patients was detrimental. Indeed, the authors showed a lower-than-expected response during ovarian stimulation in patients who received a non-individualized and fixed gonadotropin dose, which resulted in the retrieval of fewer oocytes than expected, reducing the CLBR per cycle. Finally, the study highlights the importance of correctly defining the primary endpoint when evaluating the CLBR (Leijdekkers et al., 2019).

From the patient’s perspective, it is not fair to compare CLBR over multiple cycles and conclude that the outcome is not inferior to another strategy, if the patient needs to undergo more treatment cycles to achieve the same outcome compared to a personalized strategy. The focus of the patient is to have a baby, undergoing the lowest possible number of cycles and interventions, and in the shortest period. Thus, according to the POSEIDON stratification, low prognosis should be interpreted taking into consideration the CLBR per initiated cycle (Esteves *et al*., 2019c). The above-discussed differences in reproductive outcomes and prognosis among different POSEIDON groups were explored further in two recent studies using large databases (Li *et al*., 2019; Shi *et al*., 2019).

Li *et al*. (2019) retrospectively evaluated 26,697 IVF cycles in POSEIDON group patients and calculated the optimal and conservative CLBR per cycle. The optimal estimate was based on the reported data and assumed that the CLBR in women who discontinued ART treatment without a live birth would be the same as that of women who continued treatment. In contrast, the conservative estimate assumes that those who discontinue ART treatment have a live-birth rate of zero. In POSEIDON groups 1, 2, 3, and 4, the CLBRs per initiated cycle were 56.0%, 30.1%, 14.7%, and 6.6% respectively. After three completed cycles, the optimal and conservative CLBR were 83.9% and 66.1%, 53.7% and 37.7%, and 44.2%, 28.0%, 14.2% and 9.7%, in groups 1, 2, 3, and 4, respectively (Li *et al*., 2019). In another large analysis, Shi *et al*. (2019) retrospectively evaluated 18,455 IVF cycles comparing the outcomes of the four POSEIDON groups, including non-low prognosis patients classified as patients with an AFC >=5 and a previous number of oocytes retrieved >9 oocytes (Group 5), as well as non-low prognosis patients classified as patients with an AFC >=5 and no previous OS (Group 6). The non-low prognosis patients (Groups 5 and 6) achieved a CLBR of 53.5% and 66.9%, respectively, and, thus, the highest CLBR among all groups analyzed. In contrast, POSEIDON groups 1, 2, 3, and 4 had a CLBR of 44.6%, 24.5%, 35.5%, and 12.7%, respectively (Shi *et al*., 2019).

The aforementioned results of large data analyses reveal the importance of the novel stratification proposed by the POSEIDON group, clearly showing that first of all the four groups defined by POSEIDON include patients with a poorer reproductive prognosis when compared to the normal reserve, normal responder patient; secondly, that CLBRs are different among the 4 POSEIDON groups, and thirdly, that the age of the patient is a crucial factor for the outcome of an IVF cycle; however, the ovarian response to stimulation in terms of the number of oocytes retrieved is equally important when considering the CLBR per cycle. 

### POSEIDON’s metric of success

The ability to retrieve the number of oocytes needed to obtain at least one euploid blastocyst for transfer in each patient was introduced by the POSEIDON group as a surrogate metric of success in ART (Alviggi *et al*., 2016; Humaidan *et al*., 2016). This metric was not intended to replace live birth rate (LBR), which remains the primary endpoint for couples undergoing ART. Moreover, the POSEIDON endpoint does not imply that pre-implantation genetic testing for aneuploidy (PGT-A) should be routinely performed during ART. On the contrary, it adds to the current knowledge as it provides a logical endpoint for clinicians providing care to women undergoing ART. Since the transfer of an euploid embryo provides −at any given age− implantation rates in the range of 50-60% (Forman *et al*., 2013), clinicians could plan a patient-oriented treatment with the mindset to achieve the POSEIDON metric.

### The ART calculator: a predictive tool to estimate the POSEIDON metric

**Figure 5:**
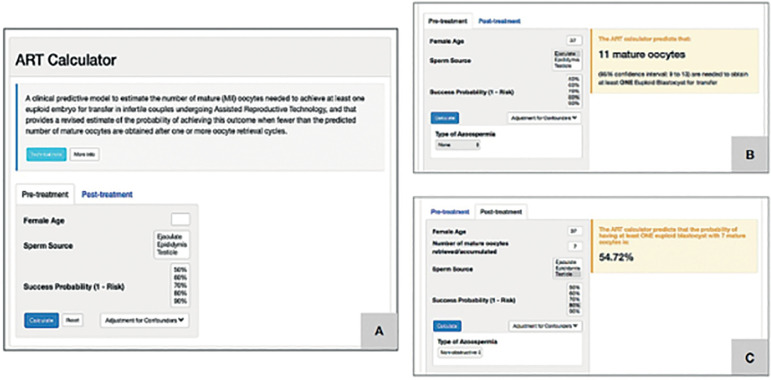
to estimate the minimum number of mature oocytes required to obtain at least one euploid blastocyst for transfer in infertile patients undergoing IVF/ICSI cycles. Reprint from: Esteves et al. A novel predictive model to estimate the number of mature oocytes required for obtaining at least one euploid blastocyst for transfer in couples undergoing in vitro fertilization/intracytoplasmic sperm injection: The ART Calculator. Front Endocrinol. 2019; 10: 99. This is an open-access article distributed under the terms of the Creative Commons Attribution License (CC BY). The online ART calculator can be found at http://www.members.groupposeidon.com/Calculator/.

The ART Calculator was developed to estimate the minimum number of metaphase II (MII) oocytes required to have at least one euploid blastocyst for transfer in patients undergoing ART (Esteves *et al*., 2019b) - Figure 5A.

This predictive tool was developed based on clinical and embryonic data of infertile couples subjected to IVF/ICSI and PGT-A. Twenty-six co-variates were analyzed, including patient demographics and treatment characteristics using the LASSO logistic regression method for variable selection. Among these co-variates, female age, type of sperm used for ICSI, and MII oocytes (*p*<0.0001) were deemed relevant for model building. The model equation provides the individualized probability of blastocyst euploidy per MII oocyte, given the female age and sperm source (and type of azoospermia), with 72% accuracy. Using mathematic equations, an online calculator was developed, in which the user can set the probability of success, i.e. the probability of having at least one euploid blastocyst when the estimated number of MII oocytes is achieved. Thus, two types of predictions can be made. Pre-treatment, the ART calculator estimates the minimum number of mature oocytes, with its associated 95% confidence interval, to achieve ≥ 1 euploid blastocyst for transfer in infertile couples undergoing IVF/ICSI. Clinicians should input the patient age and the sperm source to be used for IVF/ICSI. If the option ‘Testicle’ is marked, then the type of azoospermia should be also defined. The probability of success is set by the user and indicates the chance of having ≥1 euploid blastocyst when the predicted number of mature oocytes is achieved. Its complement is the risk, i.e., the chance of having no (zero) euploid blastocysts when the predicted number of oocytes is achieved. Once the button ‘calculate’ is pressed, a text box will pop-up on the right side of the screen, indicating the predicted minimum number of mature oocytes needed for obtaining at least one euploid blastocyst, with its 95% confidence interval (Figure 5B). Post-treatment, it provides a revised estimate of the probability of achieving ≥ 1 euploid blastocyst when fewer than the predicted number of mature oocytes are obtained after ≥ 1 oocyte retrieval cycle, i.e., when fewer than the predicted number of mature oocytes are obtained after one or more oocyte retrieval cycles, clinicians should input the pretreatment information and the actual number of mature oocytes collected or accumulated. As in the pretreatment model, the user sets the probability of success. Once the button ‘calculate’ is pressed, a text box will pop-up on the right side of the screen, indicating the predicted probability of achieving ≥1 euploid blastocyst with the number of mature oocytes available (Figure 5C). (http://www.members.groupposeidon.com/Calculator/).

A multicenter and international collaborative group was created to perform the external validation of the ART calculator. ART databases concerning infertile couples undergoing IVF/ICSI and PGT-A from three Fertility Centers (Italy, Brazil, and Turkey) were utilized. In the study, 1,464 patients, 9,779 MII oocytes, and 3,108 blastocysts were evaluated. A validation model was developed, using the same roadmap of the ART calculator. Like in the ART calculator, female age and type of sperm are relevant predictors of blastocyst euploidy. High correlations (r~0.90) were found between the outputs of the ART Calculator and validation model. Moreover, the frequency of patients in the validation dataset who (i) reached the minimum number of MII oocytes (estimated by ART Calculator), and (ii) who had at least one euploid blastocyst matched the ART Calculator output; thus, suggesting generalizability (Esteves *et al*., 2019b).

### Use of POSEIDON criteria in Clinical Practice

**Figure 6:**
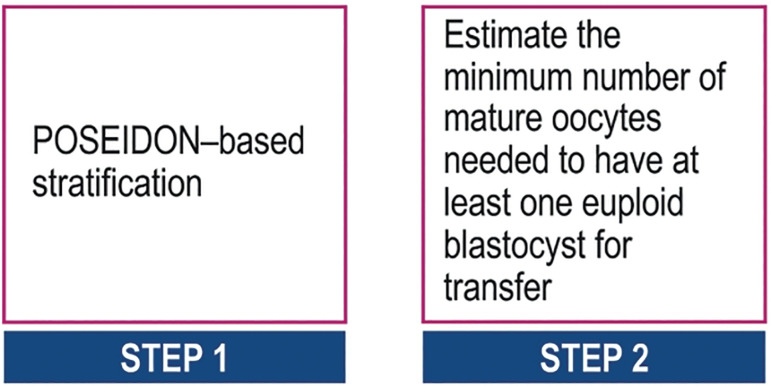
A POSEIDON-based stepwise-proposed algorithm for clinical management of patients undergoing ART

**Figure 7:**
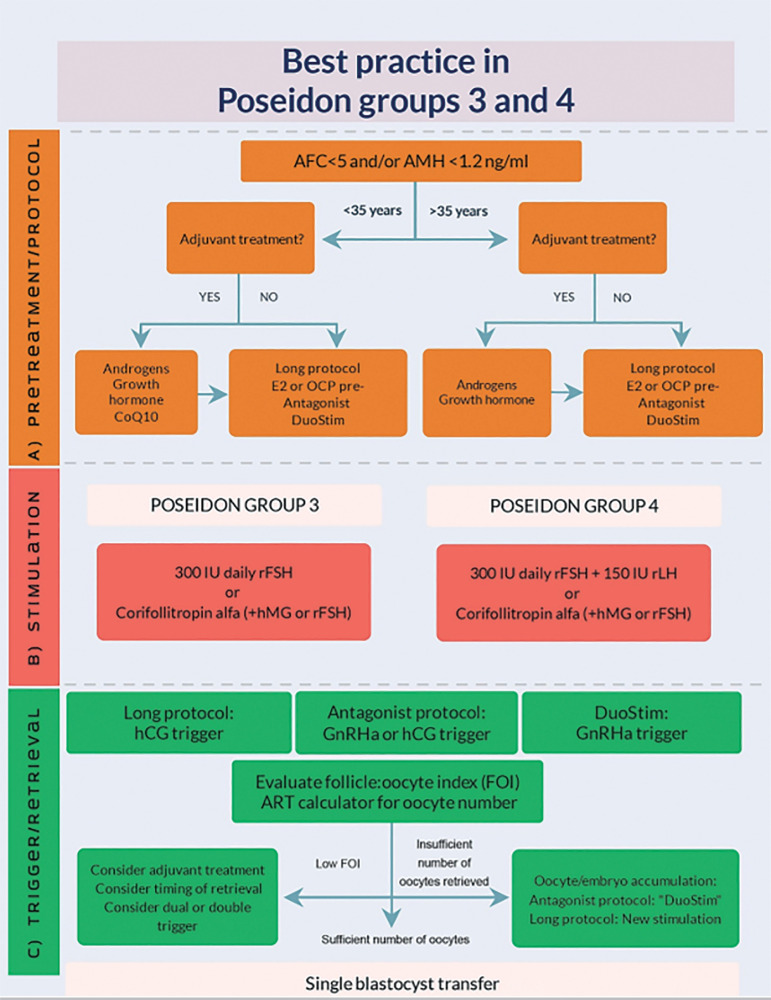
Management of POSEIDON groups 3 and 4. Reprint from: Haahr et al. Management Strategies for POSEIDON Groups 3 and 4. Front Endocrinol. 2019;10:614. This is an open-access article distributed under the terms of the Creative Commons Attribution License (CC BY).

Figure 6 depicts (http://www.members.groupposeidon.com/Calculator/) a stepwise algorithm for clinical management of patients undergoing ART. The eligible patient is first of all classified according to the POSEIDON criteria. Secondly, the minimum number of MII oocytes required to obtain at least one euploid blastocyst is estimated with the aid of the ART calculator. Lastly, patient-oriented treatment strategies to achieve the individualized oocyte number are implemented. It is out of the scope of the present review to discuss possible strategies for each POSEIDON group, however, comprehensive reviews on the subject can be found elsewhere (see also Figures 2 and 7).

## CONCLUSIONS

The POSEIDON stratification of the low prognosis patient was primarily introduced to provide a more detailed stratification of the low prognosis patient undergoing ART, with the ultimate goal of offering clinicians the guidance concerning the most suitable patient-oriented strategies to achieve the suggested oocyte number needed for one euploid blastocyst (Poseidon’s metric of success). The novel criteria, therefore, categorize patients according to their prognosis, emphasizing how female age and its related embryo aneuploidy rate, as well as oocyte number are important factors in the success of ART. Added to this, the POSEIDON criteria included the “unexpected poor/suboptimal responders” as a distinct category of “low prognosis” patients. The POSEIDON stratification is suggested to be a counseling instrument to help clinicians set the expectations of any given patient prior to OS. Moreover, the POSEIDON criteria introduced an objective measure of success for an OS cycle, namely the number of oocytes needed to obtain one euploid blastocyst for transfer. The ART calculator can be used to estimate such numbers, thus, allowing open and transparent reporting of information about the number of oocytes needed to obtain an euploid blastocyst, possibly facilitating a mature discussion about therapeutic alternatives and costs. Lastly, the POSEIDON criteria might allow selection of more homogeneous groups of patients in interventional trials, including the use of metrics, in particular, the follicle-to-oocyte index (FOI) and the number of mature oocytes needed to achieve at least one euploid blastocyst, as secondary endpoints.
